# Prevalence of HIV among healthcare workers in the post-HAART era – a systematic review and meta-analysis

**DOI:** 10.3205/dgkh000634

**Published:** 2026-03-02

**Authors:** Roland Diel, Albert Nienhaus

**Affiliations:** 1 Institute of Epidemiology, University Medical Hospital Schleswig-Holstein, Kiel, Germany; 2 Institution for Statutory Accident Insurance and Prevention in the Health and Welfare Services (BGW), Hamburg, Germany; 3 Competence Center for Epidemiology and Health Services Research for Healthcare Professionals (CVcare), Institute for Health Services Research in Dermatology and Nursing (IVDP), University Medical Center Hamburg-Eppendorf (UKE), Hamburg, Germany

**Keywords:** HIV, healthcare workers, occupational exposure, prevalence, highly active antiretroviral therapy, HAART, meta-analysis

## Abstract

**Background:**

Healthcare workers (HCWs) are at potential risk of HIV infection through occupational exposure. However, with the advent of highly active antiretroviral therapy (HAART), universal precautions, and post-exposure prophylaxis, the global risk profile has changed.

**Objective:**

This study synthesized data on HIV prevalence among HCWs worldwide to assess current epidemiological patterns and to evaluate regional heterogeneity.

**Methods:**

Following PRISMA guidelines, we systematically searched PubMed, Scopus, and the Cochrane Library for studies reporting serological evidence of HIV infection among HCWs published between 1996 and September 2025, corresponding to the post-HAART era. Data were extracted on study design, sample size, and the number of HIV-positive cases. Prevalence estimates were compared with internal control groups or with national background prevalence data obtained from UNAIDS. Methodological quality was assessed using the Westermann score. Random-effects meta-analyses were performed to estimate pooled HIV prevalence by WHO region.

**Results:**

Of 220 studies identified, 14 from 12 countries met inclusion criteria. Across 7,705 HCWs tested, the overall pooled HIV prevalence was 0.68% (95% CI 0.19–2.46) with extreme heterogeneity (I²=94.3%, τ²=5.003). Whilst the pooled prevalence among HCWs in Sub-Saharan Africa was 7.1% (95 % CI 3.3–12.7), all other regions—Europe, Latin America, and the Eastern Mediterranean—showed pooled prevalences of 0.0% (95% CI 0.0–3.7), with no HIV-positive HCWs detected.

**Conclusions:**

HIV infection among HCWs is now exceedingly rare worldwide, with elevated prevalence confined to high-endemic African regions as a regional dichotomy. The findings indicate that HIV infection in HCWs is primarily driven by community transmission rather than occupational exposure and highlight the success of infection-control policies and ART expansion in eliminating workplace transmission. Sustained surveillance and equitable access to protective equipment and post-exposure prophylaxis remain essential to preserve this major occupational-health achievement.

## Zusammenfassung

**Hintergrund:**

Medizinisches Personal (MP) ist potenziell dem Risiko einer HIV-Infektion durch berufliche Exposition ausgesetzt. Mit der Einführung der hochaktiven antiretroviralen Therapie (HAART), der konsequenten Anwendung von Basishygienemaßnahmen sowie der Verfügbarkeit einer Postexpositionsprophylaxe hat sich das globale Risikoprofil jedoch grundlegend verändert.

**Ziel:**

Ziel dieser Studie war es, weltweit Daten zur HIV-Prävalenz bei MP zu erheben, um aktuelle epidemiologische Muster zu erfassen und regionale Unterschiede zu bewerten.

**Methode:**

In Übereinstimmung mit den PRISMA-Leitlinien wurde eine systematische Literaturrecherche in PubMed, Scopus und der Cochrane Library durchgeführt. Eingeschlossen wurden Studien, die zwischen 1996 und September 2025 – entsprechend der Post-HAART-Ära – über serologische Nachweise von HIV-Infektionen bei MP berichteten. Extrahiert wurden Angaben zum Studiendesign, zur Stichprobengröße und zur Anzahl HIV-positiver Fälle. Prävalenzschätzungen wurden mit internen Kontrollgruppen oder mit nationalen Hintergrundprävalenzen verglichen, die aus UNAIDS-Daten gewonnen wurden. Die methodische Qualität der Studien wurde mithilfe des Westermann-Scores bewertet. Zur Schätzung der gepoolten HIV-Prävalenz nach WHO-Regionen wurden Random-Effects-Metaanalysen durchgeführt.

**Ergebnisse:**

Von insgesamt 220 identifizierten Studien erfüllten 14 aus 12 Ländern die Einschlusskriterien. Bei insgesamt 7.705 getesteten Beschäftigten im Gesundheitswesen betrug die gepoolte HIV-Prävalenz 0,68% (95%-KI 0,19–2,46) mit allerdings ausgeprägter Heterogenität (I²=94,3%, τ²=5,003). Während die gepoolte Prävalenz unter MP in subsaharischen Afrika 7,1% (95%-KI 3,3–12,7) betrug, zeigten alle anderen Regionen – Europa, Lateinamerika und der östliche Mittelmeerraum – eine gepoolte Prävalenz von 0,0% (95%-KI 0,0–3,7), wobei keine HIV-positiven Beschäftigten nachgewiesen wurden.

**Schlussfolgerungen:**

HIV-Infektionen bei MP sind weltweit mittlerweile äußerst selten, wobei erhöhte Prävalenzen auf Hochendemiegebiete in Afrika beschränkt sind. Die Ergebnisse sprechen dafür, dass HIV-Infektionen bei MP überwiegend durch Transmissionen in der Allgemeinbevölkerung und nicht durch berufliche Exposition bedingt sind. Gleichzeitig unterstreichen sie den Erfolg von Infektionsschutzmaßnahmen und der breiten Verfügbarkeit antiretroviraler Therapie zur Eliminierung beruflicher HIV-Übertragungen. Eine fortgesetzte Surveillance sowie ein bedarfsgerechter Zugang zu persönlicher Schutzausrüstung und Postexpositionsprophylaxe bleiben jedoch essenziell, um diesen bedeutenden Erfolg im Arbeitsschutz dauerhaft zu sichern.

## Introduction

Globally, approximately 39 million people were living with HIV in 2023, according to the Joint United Nations Programme on HIV/AIDS (UNAIDS) and the World Health Organization (WHO), corresponding to a global prevalence of about 0.7% among adults aged 15–49 years [1], [2]. The highest burden remains concentrated in sub-Saharan Africa, where prevalence still exceeds 5% in several countries, whereas rates in high-income regions such as Europe and North America have stabilized at below 0.3% [1], [2], [3].

The overall epidemiological landscape of HIV infection has therefore shifted from an expanding global epidemic in the late twentieth century to a controlled chronic infection, particularly in industrialized countries with widespread access to antiretroviral therapy (ART) [4], [5]. Healthcare workers (HCWs), however, represent a unique subpopulation because of their occupational exposure to blood and body fluids. Before the advent of highly active antiretroviral therapy (HAART) in 1996, the potential for occupational HIV transmission was a matter of intense concern. Documented seroconversions following needlestick injuries and surgical accidents in the 1980s and early 1990s led to major anxiety within the healthcare sector and to restrictive national policies that excluded HIV-infected HCWs from performing exposure-prone procedures (EPPs) [6], [7].

Since 1996, the introduction of HAART—particularly triple-combination therapy including protease inhibitors—has fundamentally changed the natural history of HIV infection. Viral suppression achieved through effective ART virtually eliminates the risk of secondary transmission [8], [9]. This scientific breakthrough transformed both clinical management and public-health policy, shifting the focus from exclusion and risk avoidance toward evidence-based inclusion and proportionality. Nevertheless, it remains unclear how these therapeutic advances have translated into real-world epidemiological outcomes among healthcare personnel.

The actual prevalence of HIV infection among HCWs in the post-HAART era has never been systematically synthesized. Such a comprehensive global analysis is crucial to determine to which extent occupational exposure continues to contribute to infection risk and to assess whether any measurable occupational excess persists compared with general-population prevalence. Despite global progress, needlestick and sharps injuries, mucocutaneous contact, and surgical incidents continue to occur even under modern infection-control standards [10], [11], [12]. Understanding how frequently such exposures still result in HIV infection is therefore essential for maintaining an evidence-based and proportionate approach to occupational health and infection prevention.

The present systematic review considers all available studies published since 1996 that report HIV prevalence or serostatus among healthcare workers. By focusing exclusively on the post-HAART period, it seeks to clarify the magnitude of residual occupational risk in the context of effective ART and modern prevention practices. In addition, the review compares HCW data with background population prevalence, placing occupational infection risk within its broader epidemiological and public-health context.

This approach provides the first consolidated global assessment of HIV burden in healthcare personnel in nearly three decades and aims to inform future infection-control policies, risk communication, and occupational-health strategies.

## Methods

### Definition of HCWs

HCWs were defined as all medical, dental, nursing, obstetric or assisting personnel working in different areas, e.g. hospitals, outpatient clinics, doctors’ practices, dialysis facilities, nursing homes and out-patient care facilities. The decisive factor was the existence of a plausible transmission pathway within these activities.

### Literature search and study selection

A comprehensive literature search up to September 1, 2025, was conducted in PubMed, Scopus and the Cochrane library to identify reports describing the baseline prevalence of HIV infection among HCWs. The following Boolean search string was adapted for each database (“HIV” OR “Human Immunodeficiency Virus”) AND (“prevalence” OR “seroprevalence”) AND (“healthcare workers” OR “health care workers” OR “health personnel”) AND “occupational exposure” AND (1996:[Year]–2025[Year]). Search strategies were tailored to the indexing systems and functionalities of each database to maximise both sensitivity and specificity. Full search strings for both databases, together with the methodological rationale for their design, are presented in Attachment 1.

Studies providing original serological data on HIV infection in a defined healthcare worker population were eligible for inclusion. Studies limited to post-exposure or seroconversion analyses, as well as review articles, guidelines, conference abstracts, commentaries, and editorials, were excluded, along with articles whose central theme diverged from or was unrelated to HIV prevalence. No restrictions were applied regarding language, study design, subpopulation, or data-collection mode (prospective or retrospective). Reference lists of included studies and relevant reviews were manually screened to identify additional eligible publications. All records were managed using EndNote, which automatically removed duplicates. The review followed the PRISMA 2020 guidelines for systematic reviews and meta-analyses [13], [14].

### Data extraction

Two reviewers (RD and AN) independently screened titles, abstracts, and full texts, resolving discrepancies by consensus. From each study, data were extracted on the year and country of publication, study design, study period, HIV-testing method, number tested, number HIV-positive, and the presence or absence of a comparison group. Internal control groups were retained where available.

For studies without an internal comparator, HIV prevalence estimates for the corresponding country and year were derived from UNAIDS [15] or other national population-based surveys and surveillance reports, e.g. the Polish National Institute of Public Health – National Institute of Hygiene (NIZP-PIB) [16], representing the general adult population (aged 15–49 years). When studies reported multi-year periods, the UNAIDS estimate for the midpoint year was selected to ensure temporal consistency. To maintain comparability with global HIV surveillance systems, countries were grouped according to the WHO regional classification. 

### Quantitative synthesis and meta-analysis

A quantitative synthesis was performed to estimate the pooled HIV prevalence among HCWs. Given the substantial and expected heterogeneity in prevalence arising from vastly different epidemiological contexts across countries, the analysis was stratified *a priori* by geographical region (Sub-Saharan Africa, Europe, North America, Asia, and Latin America). 

Within each geographical stratum, the pooled prevalence was estimated using a random-effects model, which accounts for both within-study variance and between-study variance (τ²). Statistical analyses were performed using the metafor package (version 4.4-0) in the R statistical environment (version 4.3.1). Prevalence estimates with 95% confidence intervals for individual studies were calculated using the exact Clopper-Pearson method. Heterogeneity was quantified using the I² statistic.

### Assessment of study quality

Study quality was evaluated using the Westermann 9-item checklist which assesses methodological clarity, representativeness, and laboratory reliability [17]. Each fulfilled item scored one point (maximum=9); studies were classified as low (≤4), moderate (5–7), or high (≥8) quality]. For studies with zero HIV-positive participants, a Zero-Event-Adjusted (ZEA) scoring was applied, treating confirmatory testing as not applicable and rescaling totals to a nine-point maximum (×9/8). ZEA scores were first calculated to one decimal place and then rounded up to the next whole-number value for categorical classification. This adjustment prevents artificial downgrading of zero-event studies that could not perform confirmatory testing because no positive results were obtained, thereby maintaining comparability across all studies. Both versions (all items scored 0–1) were applied. 

## Results

### Study availability

**Figure 1:**
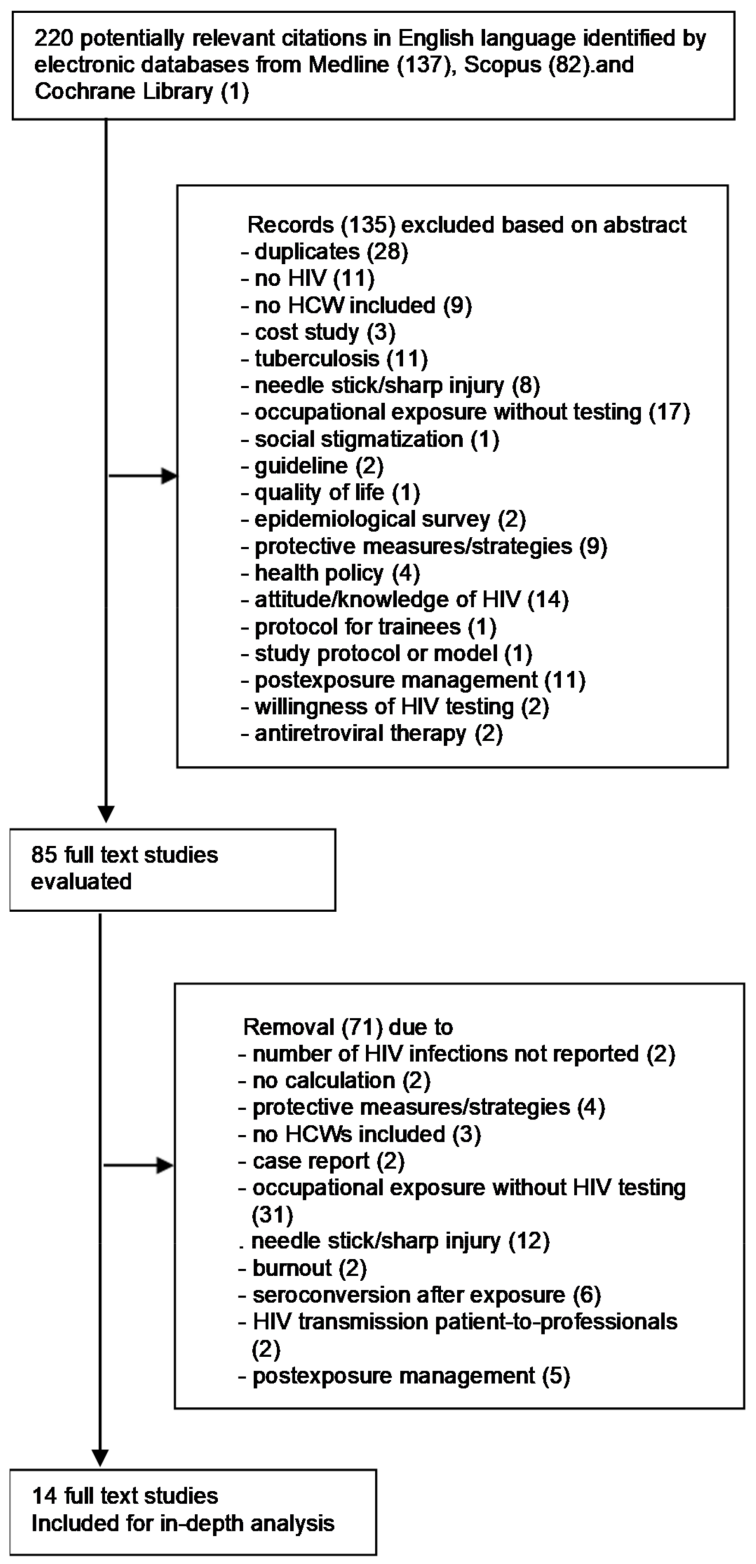
Prisma flow diagram of study selection

Figure 1 (Fig. 1) shows the flow diagram of the literature search. In total, 220 abstracts were identified (137 in PubMed, 82 in Scopus and 1 in the Cochrane Library), with reviews being excluded by default in the search strategy; 135 studies, of those 28 were duplicates, were excluded based on their abstracts. 85 full-text articles were reviewed. Of these, 14 peer-reviewed studies met the eligibility criteria [18], [19], [20],[21], [22], [23], [24], [25], [26], [27], [28], [29], [30], [31] and were included in the analysis.

### Study design

Thirteen of the 14 studies (92.9%) were cross-sectional investigations. One study (7.1%) was a prospective cohort design where blood was drawn from Dutch expatriate HCWs, on average, 30 days post-return. 

### Study characteristics

The characteristics of the included studies are summarized in Attachment 2, Tab. 1 . All 14 studies were published between 1998 and 2025 and came from 12 different countries. Two studies each were conducted in South Africa and Poland (2/14 or 14.3%), while Brazil, Burkina Faso, Cameroon, Denmark, Georgia, Iraq, Mozambique, the Netherlands, Turkey and Pakistan each contributed one (1/14 or 7.1%). Study sample sizes ranged from 99 to 1,386 participants.

The total number of HCWs serologically tested for HIV antibodies across all studies was 7,705, with 179 HIV-positive individuals. The crude (unweighted) aggregated prevalence representing the simple overall proportion of HIV-positive cases was therefore 2.32% (95% CI 2.00–2.68%, Clopper–Pearson exact). The global random-effects pooled prevalence, however, was 0.68%, (95% CI 0.19–2.46%) and reflects the high weighting influence of numerous zero-event studies outside Sub-Saharan Africa and the extreme inter-regional heterogeneity (I²=94.3%, τ²=5.003). Table 1 (Tab. 1) summarizes the regional distribution and corresponding pooled prevalences.

Stratified by WHO regions, all five European studies reported zero HIV-positive cases (0/3462), yielding a pooled prevalence of 0.0% (95% CI: 0.0–0.17%) and no heterogeneity (I²=0%,τ²=0), indicating highly consistent findings across settings in Europe. Similarly, in the Eastern Mediterranean region (Pakistan and Iraq), two cross-sectional studies screened a total of 1 426 HCWs, detecting no HIV-positive cases and yielding a pooled prevalence of 0.0% (95% CI: 0.0–0.2%). No heterogeneity was detected (I²=0%, τ²=0). This consistency across Pakistan and Iraq supports the inference of negligible occupational HIV risk for HCWs also in these low-prevalence settings. Latin American region (Brazil) was represented by a single study from Brazil with no HIV-positive HCW (prevalence of 0.0%; 95% CI: 0.0-0.9), so no meta-analysis was possible. 

In contrast, substantial heterogeneity was observed across the five African studies (I²=95.7%), with individual prevalence estimates ranging from 2.6% to 27.4%. The highest proportions originated from southern Africa (South Africa and Mozambique), while studies from West and Central Africa (Burkina Faso, Cameroon) reported considerably lower rates (2–4 %), consistent with background population prevalence levels. The pooled random-effects estimate yielded an overall HIV prevalence of 7.1% (95% CI: 3.3–12.7%), however, the wide confidence interval and the high I² of 95.7% reflect marked variability in prevalence across countries and years. Thus, the pooled estimate for HIV prevalence among African HCWs should be interpreted as an average of widely varying national prevalences rather than a single, true prevalence.

### Summary of methodological quality

The methodological quality of the 14 included studies was evaluated using the nine-item Westermann checklist [17]. Under strict scoring, total scores ranged from 4 to 9 points (median=7). Most studies (11/14; 79%%) were rated as moderate quality (5–7 points), one as low quality (=4 points), and two as high quality (=8 points).

To account for studies reporting zero HIV-positive participants, a Zero-Event-Adjusted (ZEA) scoring was applied, in which the confirmatory-test criterion was treated as not applicable, and totals were rescaled to a nine-point maximum (×9/8). ZEA scoring slightly increased scores for zero-event studies (mean +0.6 points), but only one study changed quality category (Attachment 2).

### Comparison with internal and external control groups

In this review, UNAIDS estimates and other population-based surveillance data were used to determine whether observed HIV prevalence among HCWs aligned with, exceeded, or fell below that of the general adult population during the same period and within the same country. In nearly all cases—with exception of two African datasets, those of Kirakoya-Samadoulougou et al. in Burkina Faso [25] and Casas et al. in Mozambique [21]—HCW prevalence equaled or was lower than national background levels. This pattern indicates that HIV infections among HCWs predominantly reflect community-level epidemiology rather than occupational transmission.

The two outlier studies showing higher HIV prevalence were both substantially affected by selection bias, which likely inflated the observed infection rates. In the study by Casas et al. [21], an extraordinarily high overall HIV prevalence of 43.8% among hospital staff was reported. However, only 47.5% of the workforce participated, almost certainly attracting individuals already aware of their HIV status or perceiving themselves at higher risk. This bias persisted even within the subset of 113 previously untested HCWs, among whom 31 new infections (27.4%) were identified. Because testing was voluntary and non-anonymous, this subgroup was also not representative, and the reported prevalence likely overestimates the true infection rate among the hospital’s total staff. Similarly, the national survey by Kirakoya-Samadoulougou et al. [25] suffered from participation bias. Although the overall prevalence was clearly lower (3.5%), the testing acceptance rate was only 64.5%, and medical doctors constituted merely 1% of participants, rendering the sample non-representative. Comparison with general population data is further complicated by demographic differences: the HCW cohort was older and more urbanized, both independent risk factors for HIV. After statistical adjustment for these confounders, the HIV prevalence among male HCWs (2.5%) became nearly identical to that of men in the general Demographic and Health Surveys (DHS) population (2.3%), demonstrating that the apparent excess risk was attributable to demographic composition rather than occupation. Finally, Fisker et al. [19] provided the only dataset including an internal comparison group of non-HCWs from the same population (0/539; 95% CI 0–0.68%). Their results mirrored the background epidemic level in Denmark, further supporting the absence of occupationally related excess risk.

## Discussion

To our knowledge, this analysis represents the first systematic review of published reports on HIV prevalence among HCWs since the introduction of HAART. Across 14 studies with serological test results spanning 28 years (1998–2025), only 179 HIV infections were identified among a total of 7,606 HCWs tested. Although a crude global HIV prevalence of 2.38% suggests that infection among HCWs is rare, this aggregate figure masks profound geographical disparities.

HIV prevalence among HCWs shows a strikingly regional pattern, statistically confirming a pronounced divide. Elevated HCW prevalence is observed only in the context of generalized epidemics in sub-Saharan Africa, where prevalence remains substantial and highly heterogeneous. These figures mirror the broader regional epidemic and reflect the diversity of national HIV contexts. In contrast, in Europe, North America, Asia, and Latin America, HIV prevalence among HCWs is statistically indistinguishable from zero, with pooled estimates consistently at 0% and — depending on study size — extremely narrow upper confidence limits. This provides robust quantitative evidence that occupational HIV transmission risk is negligible in low-prevalence settings.

This interpretation is supported by the landmark study of de Graaf et al. [18], which effectively served as a natural experiment. The study examined a cohort of Dutch expatriate HCWs working in AIDS-endemic countries — individuals with high occupational exposure risk but originating from a low-prevalence population. The finding of zero seroconversions despite frequent needlestick injuries clearly demonstrates that the infection risk profile of the population of origin is a stronger determinant of HIV acquisition than occupational exposure itself.

The pronounced heterogeneity observed within the African stratum (I²>95%) confirms that HCWs cannot be regarded as a uniform risk group. Their infection risk largely mirrors the background HIV prevalence of the communities in which they live and work, rather than occupational exposure. In addition to this statistical heterogeneity, there is a near-complete absence of any measurable occupational excess risk. When benchmarked against UNAIDS or national surveillance estimates, HIV prevalence among HCWs is consistently equal to or lower than that of the general population across all WHO regions. This pattern supports the conclusion that, in the HAART era and under effective infection-control standards, the occupational transmission risk of HIV in healthcare settings is negligible.

The only two apparent outliers — studies from Mozambique [21] and Burkina Faso [25] — reported comparatively elevated HIV prevalence among HCWs, but these findings are likely inflated by methodological bias, particularly self-selection of participants. In the Burkina Faso study, the apparent excess risk among male HCWs disappeared after adjustment for confounders such as age and place of residence, indicating that demographic rather than occupational factors explained the observed differences. These methodological limitations likely account for the higher HCW prevalence compared with the surrounding general population and underscore the importance of representative sampling and independent testing frameworks in occupational-health surveillance. Consequently, these skewed prevalence figures should not be interpreted as representative benchmarks for occupational risk among African HCWs.

In summary, the pronounced geographical dichotomy — where HCW HIV prevalence is virtually zero in low-prevalence regions and rises in parallel with community prevalence in high-burden countries — strongly indicates that community acquisition, not occupational exposure, is the dominant pathway of infection in this professional group. While occupational risk cannot be considered absolutely zero, its contribution to overall infection burden is negligible compared with the influence of background community transmission.

Consistently near-zero prevalence in Europe, the Americas, and Asia underscores the effectiveness of infection-control practices, personal protective equipment (PPE), and post-exposure prophylaxis (PEP). This conclusion is further supported by longitudinal CDC surveillance data [32], which documented a sharp decline in occupationally acquired HIV in the United States: 58 confirmed and 150 possible cases were reported between 1985 and 2013, but only one confirmed case since 1999. The CDC attributed this success to the widespread adoption of standard precautions, engineered sharps protection, and PEP availability in the HAART era.

These findings reinforce the rationale for modern, non-restrictive, U=U-aligned guidelines (“Undetectable=Untransmittable”), such as the UK Advisory Panel for Healthcare Workers Living with Bloodborne Viruses (UKAP) 2024 [33] and the Society for Healthcare Epidemiology of America (SHEA) 2022 [34] recommendations, which permit virally suppressed HCWs to perform all procedures while ensuring equitable access to prevention and treatment resources in under-resourced settings.

Nevertheless, sustained vigilance is warranted — particularly in high-burden African settings — where structural barriers, limited resources, and inconsistent access to protective equipment persist. Strengthening surveillance systems and maintaining investment in infection-control capacity are essential to ensure that the near-elimination of occupational HIV transmission remains a durable public-health achievement.

## Conclusions

Together with CDC surveillance data, our findings demonstrate that occupational HIV transmission among HCWs has become exceedingly uncommon since the HAART era and that infection rates largely mirror background epidemics within their respective regions. Maintaining universal precautions, ensuring timely access to PEP, and sustaining early ART coverage remain essential pillars of occupational HIV prevention.

However, the residual heterogeneity observed in sub-Saharan Africa underscores persistent challenges, including resource limitations, incomplete PPE coverage, and a higher community burden. Sustained investment in infection-control infrastructure and continued monitoring of occupational exposures are crucial to consolidate these achievements and ensure the permanent elimination of occupational HIV transmission.

## Notes

### Author’s ORCID

- Roland Diel: 0000-0001-8304-7709
- Albert Nienhaus: 0000-0003-1881-7302

### Competing interests

The authors declare that they have no competing interests.

## Supplementary Material

Search strategies

Quality assessment

## Figures and Tables

**Table 1:**
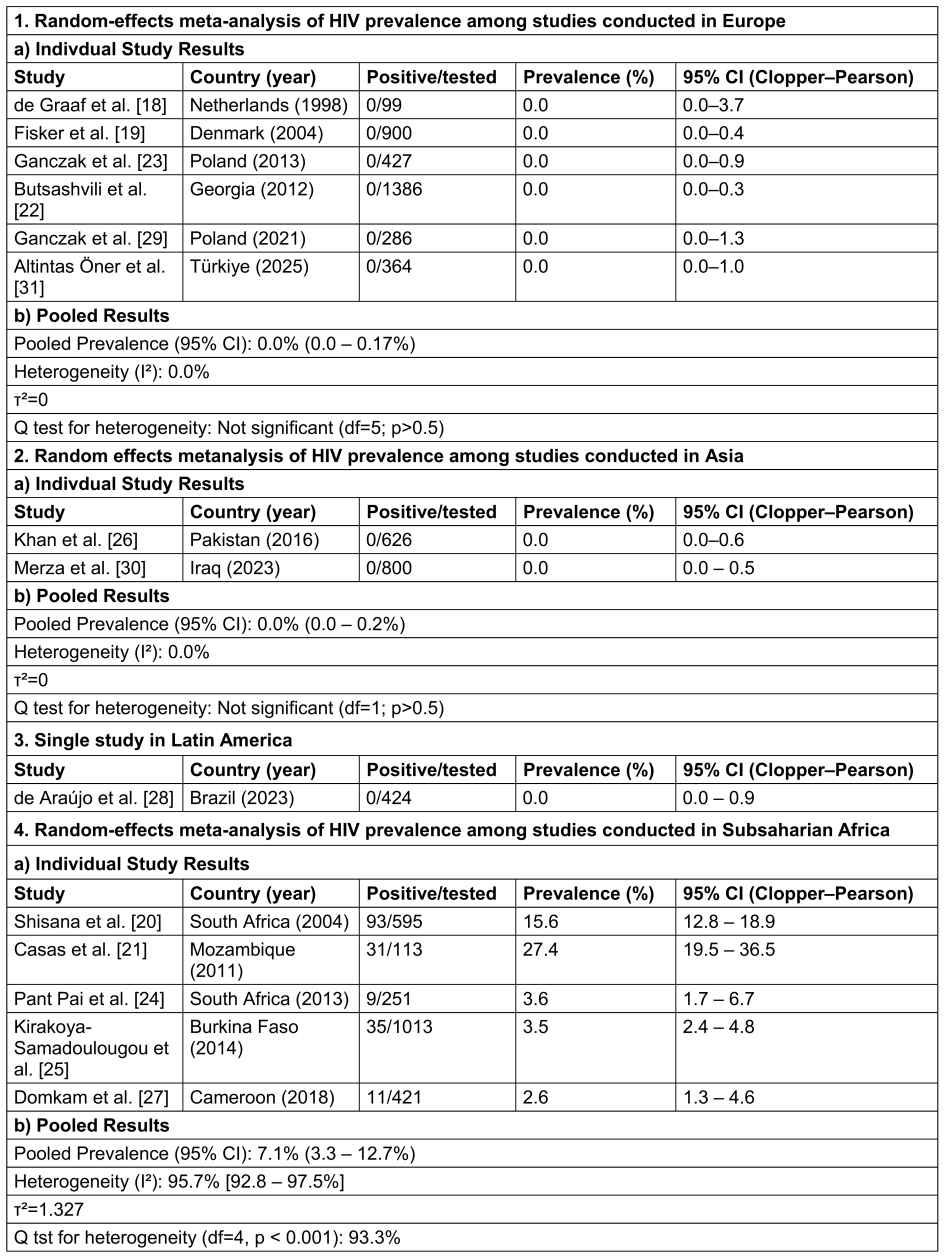
Pooled HIV prevalences among healthcare workers, stratified by region
