# Supplementary material for: Prevalence of HIV among healthcare workers in the post-HAART era – a systematic review and meta-analysis
Source: GMS Hyg Infect Control. 2026 Mar 2;21:Doc25. doi: 10.3205/dgkh000634 (PMC13103531; doi:10.3205/dgkh000634)
Supplement: Search strategies [file HIC-21-25-s-001.pdf]

Attachment 1
Search strategies

The search was performed in both PubMed and Scopus to ensure comprehensive coverage. The Scopus strategy was adapted from the PubMed strategy using database-specific syntax while maintaining the same conceptual framework.

a) PubMed Search Strategy

Text (("HIV"[Mesh] OR "HIV Infections"[Mesh]) AND 1996:2025[pdat] AND

("Occupational Exposure"[Mesh] OR occupational[tiab] OR needlestick[tiab] OR

"needle-stick"[tiab])

AND

("Health Personnel"[Mesh] OR HCW[tiab] OR "healthcare worker"[tiab] OR "health

care worker"[tiab])

AND ("prevalence"[MH] OR "Seroepidemiologic Studies"[MH] OR "Incidence"[MH] OR

prevalence[tiab] OR seroprevalence[tiab] OR incidence[tiab]))

NOT

("Review"[pt] OR "Systematic Review"[pt] OR "Meta-Analysis"[pt])

Rationale for Search Strategy Components:

1. **HIV Concept** ("HIV"[Mesh] OR "HIV Infections"[Mesh]):
This core component uses the precise Medical Subject Headings (MeSH) to ensure all studies relevant to HIV infection are captured.
2. **Date Restriction** (1996:2025[pdat]):
The year 1996 marks the advent of Highly Active Antiretroviral Therapy (HAART), which fundamentally altered the natural history of HIV.
3. **Occupational Context** ("Occupational Exposure"[Mesh] OR occupational[tiab] OR needlestick[tiab] OR "needle-stick"[tiab]):
This set of terms was designed to capture the occupational risk setting. While "Occupational Exposure"[Mesh] is the controlled vocabulary term, it was supplemented with the broader text word occupational[tiab] and the highly specific terms needlestick[tiab] OR "needle-stick"[tiab] to include studies focusing on the primary mechanism of exposure for HCWs that might not be indexed with the specific MeSH term.
4. **Population** ("Health Personnel"[Mesh] OR HCW[tiab] OR "healthcare worker"[tiab] OR "health care worker"[tiab]):
This combination ensures a comprehensive capture of the target population. The MeSH term "Health Personnel" is broad and inclusive, while the text-word searches

capture common abbreviations (HCW) and spelling variations (healthcare worker, health care worker) found in titles and abstracts.

5. **Outcome and Study Design** ("prevalence"[MH] OR "Seroepidemiologic Studies"[MH] OR "Incidence"[MH] OR prevalence[tiab] OR seroprevalence[tiab] OR incidence[tiab]): It includes both MeSH terms and text words for **prevalence** (the proportion of a population with HIV at a specific time) and **incidence** (the rate of new HIV infections over a period). The inclusion of "Seroepidemiologic Studies"[MH] specifically targets field-based studies that measure disease prevalence in populations through serological testing.
6. **Exclusion of Review Articles** (NOT ("Review"[pt] OR "Systematic Review"[pt] OR "Meta-Analysis"[pt])):
   This filter was applied to remove secondary literature, ensuring the search results prioritize primary research studies that contain original data for extraction and synthesis.

b) **Scopus Search Strategy**

(TITLE-ABS-KEY (hiv OR "human immunodeficiency virus"))

AND

(PUBYEAR>1995 AND PUBYEAR < 2026)

AND

(TITLE-ABS-KEY ("occupational exposure" OR occupational OR needlestick OR "needle-stick"))

AND

(TITLE-ABS-KEY ("health personnel" OR hcw OR "healthcare worker" OR "health care worker"))

AND

(TITLE-ABS-KEY (prevalence OR seroprevalence OR incidence OR "seroepidemiologic"))

AND

NOT (TITLE-ABS-KEY ("review" OR "systematic review" OR "meta-analysis"))
