# Supplementary material for: Prevalence of HIV among healthcare workers in the post-HAART era – a systematic review and meta-analysis
Source: GMS Hyg Infect Control. 2026 Mar 2;21:Doc25. doi: 10.3205/dgkh000634 (PMC13103531; doi:10.3205/dgkh000634)
Supplement: Quality assessment [file HIC-21-25-s-002.pdf]

Attachment 2

Application of the Westermann et al. [17] Quality Assessment Instrument to 14 HIV Prevalence Studies among HCWs. This appendix applies the Westermann et al. (Occup Environ Med 2015;72:880–888, Table 1 [17]) nine-item quality assessment checklist—originally developed for hepatitis C virus (HCV) prevalence studies—to the 11 HIV prevalence or exposure studies included in this review.

Quality assessment

The methodological quality of each included study was assessed using the nine-item Westermann checklist [17] which evaluates study design and internal validity.

The checklist includes the following criteria: (1) clearly defined study aim; (2) sample size>50; (3) response rate>50 %; (4) occupational detail; (5) inclusion of a control group; (6) adjustment for confounders; (7) statement of study limitations; (8) specification of laboratory test used; and (9) confirmatory testing.

Each fulfilled criterion was scored with one point, for a maximum of nine points. Studies were categorized as low quality (≤ 4), moderate quality (5–7), or high quality (≥ 8).

For studies that reported zero HIV-positive healthcare workers, a Zero-Event-Adjusted (ZEA) scoring was applied.

In this adaptation, the confirmatory test criterion (item 9) was treated as not applicable (NA), and the total score was rescaled to a nine-point maximum (× 9/8). ZEA scores were rounded using standard rounding conventions.

This adjustment prevents artificial downgrading of zero-event studies that could not perform confirmatory testing because no positive results were obtained, thereby maintaining comparability across all studies.

Two scoring variants were implemented for transparency:

1. Strict Westermann scoring (Table 1): All nine items were scored exactly as in the original checklist. Item 9 (Confirmatory test performed) was assigned one point only when confirmatory testing (Western blot, PCR, or second ELISA) was explicitly reported. For studies reporting zero reactive HIV tests, Item 9 was still scored as 0 (not fulfilled).

2. Zero-Event-Adjusted (ZEA) scoring (Table 2): For studies reporting no positive HIV test ("zero-event"), item 9 was coded as not applicable (NA) and excluded from the denominator (8 instead of 9 total items) and the sum of the other 8 items is then rescaled to a 9-point maximum. The total score was then rescaled to the 0–9 scale using the formula:

$$\mathrm{ZEA} = \left(\sum_{i=1}^{8} \mathrm{Item}_i \right) \times \frac{9}{8}$$

This maintains comparability to the 9-point scale used for all other studies. For studies with at least one reactive case, Item 9 was scored strictly. This modification prevents penalizing studies where no confirmatory testing was required.

Quality categories follow Westermann et al.:

8–9 = High quality 5–7 = Moderate quality ≤ 4 = Low quality.

Table 1: Westermann Scoring (Original 9 Items)

Study (Country, Year of publication)	1 Aim	2 N>50	3 Response >50%	4 Employment length	5 Control tested	6 Confounders adjusted	7 Limitations	8 Laboratory test	9 Confirmatory test	Total	Category
de Graaf et al. Netherlands, 1998 [18]	1	0	1	1	0	0	1	1	1	6	Moderate
Fisker et al., Denmark, 2004, [19]	1	1	1	1	1	1	1	1	1	9	High
Shisana et al., South Africa, 2004 [20]	1	1	1	0	0	0	1	1	NA	5	Moderate
Casas et al., Mozambique, 2011[21]	1	1	0	0	0	0	1	1	NA	4	Low
Butsashvili et al., Georgia, 2012 [22]	1	1	1	0	0	0	1	1	1	6	Moderate
Ganczak et al, Poland, 2013 [23]	1	1	1	1	0	0	1	1	NA	6	Moderate
Pant Pai et al., South Africa, 2013 [24]	1	1	1	0	0	0	1	1	1	6	Moderate
Kirakoya-Samadoulougou et al., Burkina Faso, 2014 [25]	1	1	1	1	0	1	1	1	1	8	High
Khan et al., Pakistan, 2016 [26]	1	1	1	0	0	0	1	1	NA	5	Moderate
Domkam et al., Cameroon, 2018 [27]	1	1	1	0	0	0	0	1	1	5	Moderate
de Araújo et al., Brazil, 2023 [28]	1	1	1	1	0	0	1	1	NA	6	Moderate
Ganczak et al., Poland, 2020 [29]	1	1	1	1	0	0	1	1	NA	6	Moderate
Merza et al., Iraq, 2023 [30]	1	1	1	0	0	0	1	1	NA	5	Moderate
Altıntaş Öner et al., Turkiye, 2025 [31]	1	1	1	0	0	0	0	1	1	5	Moderate

Table 2: Zero-Event-Adjusted (ZEA) scoring[1]

Study (Country, Year of publication)	Zero-event?	ZEA total	ZEA total rounded	Category
de Graaf et al., Netherlands, 1998 [18]	No	6.0	6	Moderate
Fisker et al., Denmark, 2004 [19]	No	9.0	9	High
Shisana et al., South Africa, 2004 [20]	Yes	5.6	6	Moderate
Casas et al., Mozambique, 2010 [21]	Yes	4.5	5	Moderate
Butsashvili et al., Georgia, 2007 [22]	No	6.0	6	Moderate
Gańczak et al., Poland, 2012 [23]	Yes	6.8	7	Moderate
Pant Pai et al., South Africa, 2013 [24]	No	6.0	6	Moderate
Kirakoya et al., Burkina Faso, 2014 [25]	No	8.0	8	High
Khan et al., Pakistan, 2016 [26]	Yes	5.6	6	Moderate
Domkam et al., Cameroon, 2018 [27]	No	5.0	5	Moderate
de Araújo et al., Brazil, 2015 [28]	Yes	6.8	7	Moderate
Gańczak et al., Poland, 2021 [29]	Yes	6.8	7	Moderate
Merza et al., Iraq, 2023 [30]	Yes	5.6	6	Moderate
Altintas Öner et al., Turkiye, 2025 [31]	No	5.0	5	Moderate

[1]ZEA scoring treats confirmatory testing as not applicable for studies reporting zero HIV-positive cases; the eight scored items are rescaled to a nine-point maximum (× 9/8).
